# Assessing Cadmium and Chromium Concentrations in Drinking Water to Predict Health Risk in Malaysia

**DOI:** 10.3390/ijerph17082966

**Published:** 2020-04-24

**Authors:** Minhaz Farid Ahmed, Mazlin Bin Mokhtar

**Affiliations:** Institute for Environment and Development (LESTARI), Universiti Kebangsaan Malaysia (UKM), UKM Bangi, Selangor 43600, Malaysia; minhaz@ukm.edu.my

**Keywords:** Malaysia, Langat River Basin, drinking water, hazard quotient, lifetime cancer risk

## Abstract

Although toxic Cd (cadmium) and Cr (chromium) in the aquatic environment are mainly from natural sources, human activities have increased their concentrations. Several studies have reported higher concentrations of Cd and Cr in the aquatic environment of Malaysia; however, the association between metal ingestion via drinking water and human health risk has not been established. This study collected water samples from four stages of the drinking water supply chain at Langat River Basin, Malaysia in 2015 to analyze the samples by inductivity coupled plasma mass spectrometry. Mean concentrations of Cd and Cr and the time-series river data (2004–2014) of these metals were significantly within the safe limit of drinking water quality standard proposed by the Ministry of Health Malaysia and the World Health Organization. Hazard quotient (HQ) and lifetime cancer risk (LCR) values of Cd and Cr in 2015 and 2020 also indicate no significant human health risk of its ingestion via drinking water. Additionally, management of pollution sources in the Langat Basin from 2004 to 2015 decreased Cr concentration in 2020 on the basis of autoregression moving average. Although Cd and Cr concentrations were found to be within the safe limits at Langat Basin, high concentrations of these metals have been found in household tap water, especially due to the contamination in the water distribution pipeline. Therefore, a two-layer water filtration system should be introduced in the basin to achieve the United Nations Sustainable Development Goals (SDGs) 2030 agenda of a better and more sustainable future for all, especially via SDG 6 of supplying safe drinking water at the household level.

## 1. Introduction

Cadmium (Cd) and Chromium (Cr) in the aquatic environment are mainly from the erosion of natural deposits [1,2], but can also be a result of discharge from metal refineries and runoff from waste batteries and paints [3,4,5,6]. The detrimental impact of toxic Cd and Cr on living organisms in the aquatic environment is due to their prolonged persistence and non-biodegradable characteristics [7,8]. Therefore, these metals have been listed as the toxic trace metals by the United States Environmental Protection Agency (USEPA) if ingested via drinking water. The natural weathering of mineral rocks is considered as the main source of Cd and Cr in the Langat River, Malaysia [9,10]. Low concentration of dissolved Cd (1 × 10^−3^ mg/L) was reported by Mamun et al. [11] in the Langat River; however, Sarmani [12] and Yusuf [13] observed very high concentration of Cd in Langat River (35.56 × 10^−3^ mg/L and 24 × 10^−3^ mg/L, respectively). The high concentration of Cd in Langat River might be because of sampling locations near ex-mining sites and runoff from infrastructure development activities within the Langat Basin. Similarly, Wang et al. [14] found high dissolved Cd concentration (61.74 × 10^−2^ ± 90.12 × 10^−2^ mg/L) in the Huaihe River, China. Aris et al. [9] reported a low Cr concentration (6.7 × 10^−4^ ± 9 × 10^−4^ mg/L) in the Langat River; however, higher Cr concentration was recorded (7 × 10^−2^ mg/L) in Langat River [13]. Islam et al. [7] also reported high Cr concentration (7.8 × 10^−2^ ± 2.7 × 10^−4^ mg/L) in the Korotoa River, Bangladesh.

The corrosion of galvanised pipes is also attributed to higher concentration of Cd and Cr in the tap/drinking water globally, including in Malaysia [15,16]. For instance, Ong et al. [17] reported Cd concentrations of 1.33 × 10^−3^ mg/L and Cr 1.24 × 10^−3^ mg/L in the tap water of Kuala Lumpur, Malaysia. Similarly, Nalatambi [16] found the concentrations of Cd to be 1.3 × 10^−5^ mg/L and Cr 6.75 × 10^−3^ mg/L in the tap water at Sunway Kuala Lumpur, Malaysia. Low levels of Cd (4.1 × 10^−5^ ± 1 × 10^−5^ mg/L [18] and 2 × 10^−5^ ± 1 × 10^−5^ mg/L [19]) and Cr (8 × 10^−3^ ± 4 × 10^−4^ mg/L [19]) have also been reported in the Langat River Basin, Malaysia. Langat River is one of the main sources of drinking water in the State of Selangor, Malaysia, providing drinking water to almost one-third of the population in the state [20,21,22]. However, no studies in Malaysia have linked the association between Cd and Cr ingestion via drinking water and human health risk.

The association between Cd exposure and renal cancer has been reported by studies in Thailand [23], China [24], the USA [25,26,27], and Europe [28,29,30]. Cadmium is highly toxic and highly soluble in water [31,32], so even at low levels (i.e., 1 × 10^−3^ mg/L) the ingestion of cadmium via drinking water may cause acute gastroenteritis [33], renal tubular dysfunction, and renal cancer [34,35], as well as histopathological changes in humans [36]. Therefore, humans are very susceptible to the acute toxicity of Cd ingestion via drinking water because of its 10–35 year biological half-life [37,38] as well as its bioavailability and bio accumulative characteristics [27,39]. Hence, Cd is classified as a human carcinogenic (Group 2A) by the International Agency for Research on Cancer [31] and the European Commission [40] on the basis of human and animal experiments. In addition to carcinogenic impacts of Cd exposure, non-carcinogenic impacts such as chronic kidney disease (CKD), hypertension, diabetes, bone defects, and macular degeneration have been observed [23,24,25,26,27,28,29,30,31,32,33,34,35,36,37,38,39,40,41,42,43,44].

On the other hand, Cr is naturally found in environmental media originating both from ingenious geologic formations and anthropogenic activities [2,45]. Chromium is also highly soluble in water and has prolonged persistence in the environment [46]. Therefore, both the IARC (International Agency for Research on Cancer) and USEPA have classified Cr (VI) as a group 1 human carcinogen because of its acute toxicity via ingestion [47,48,49]. The carcinogenic characteristics of Cr (VI) are based on laboratory experiments on animal stomachs, intestinal tracts, and lung [50,51]. Therefore, human epidemiology is required to find out the association between Cr ingestion via drinking water and various forms of cancer [52,53]. Meanwhile, several studies have reported the association between the lung cancer of workers in the USA and the inhalation of Cr (VI) [54]. The U.S. National Institute for Occupational Safety and Health has estimated that the lifetime risk of lung cancer death at exposure to 1 × 10^−6^ mg/L Cr (VI) is 6 per 1000 workers; exposure to 2 × 10^−7^ mg/L Cr (VI) has been estimated to be approximately 1 lung cancer death per 1000 workers [55]. Similarly, Cr (VI) was detected in about one-third of 7000 drinking water sources surveyed by the State of California in the USA (detection limit 1 × 10^−3^ mg/L), although the reported concentrations of Cr (VI) was relatively low (86% < 1 × 10^−2^ mg/L) [56]. It is suspected that about 200 million people in USA across all the 50 states have been exposed to higher than recommended levels of Cr (VI) through their tap water; therefore, they are susceptible to more than 12,000 new cases of cancer in 2014 [57,58]. Beaumont et al. [56] reported that ingestion of Cr (VI) via drinking water is possibly associated with stomach cancer in China. Non-carcinogenic risks of Cr (VI) exposure include diarrhea, stomach and intestinal bleeding, cramps, liver damage, and kidney damage [59,60]. This study determined the Cd and Cr status in the drinking water supply chain at the Langat River Basin, Malaysia. Additionally, predictions are made on the potential human health risk of Cd and Cr ingestion to suggest better management of drinking water. 

## 2. Materials and Methods

### 2.1. Water Quality Determination 

**Figure 1:**
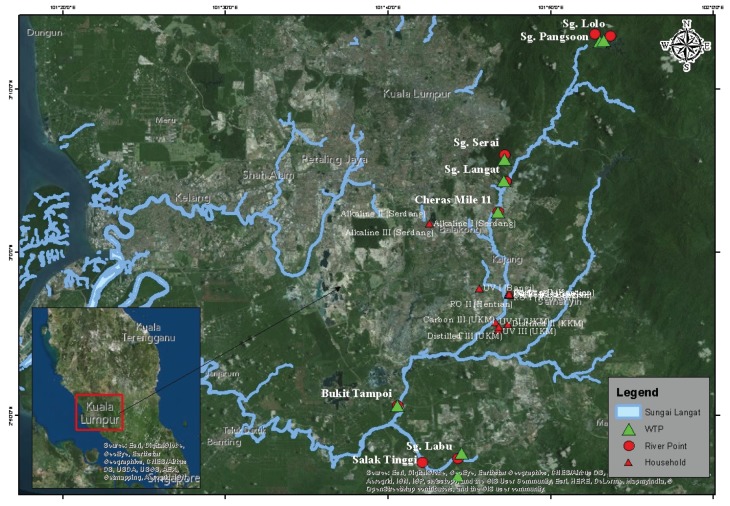
Water sampling locations in the Langat River Basin, Malaysia.

Water samples were collected one time in 2015 from the four stages of drinking water supply chain (i.e., river water, water treatment plant (WTP), household (HH) tap water, and post- filtration water) at Langat River Basin, Malaysia. Three replicates of water samples were collected from the eight points of Langat River where the WTPs collect water for drinking water treatment purposes. Three replicates of water samples were also collected from the outlets of the eight WTPs. Three replicates of household tap water and post-filtration filtered water samples were also collected on the basis of the five types of water filtration systems in the same households (Figure 1). A Chelex 100 resin column ion-exchange method was applied to analyze the dissolved Cd and Cr concentrations in the water samples [61,62] by the inductive coupled plasma mass spectrometry (ICP-MS). Standards of several concentrations were prepared to calibrate the analysis of these metals by ICP-MS. Blanks were also prepared to avoid the error in the results of metal concentrations. Multi-element calibration standard III (PerkinElmer, Lot #CL7-173YPY1, PE #N9300233) was used for the recoveries of the standard reference material (SRM); it was calculated for Cd at 94.966% ± 0.295% and Cr at 99.803% ± 0.005%. ANOVA was performed using SPSS software (IBM Corp., Armonk, NY, USA, Version 21.0) to compare Cd and Cr concentrations among the four stages of drinking water supply chain and among the sampling locations in the Langat River Basin. 

### 2.2. Human Health Risk Assessment 

The USEPA has listed Cd and Cr as highly toxic contaminants that can have cancer risks if ingested for a long period of time [32,47,63]. Therefore, to assess the human health risk, the USEPA established a model of chronic daily intake (CDI) of chemicals [64], non-carcinogenic hazard quotient (HQ), and carcinogenic lifetime cancer risk (LCR) [3] based on Cd and Cr ingestion via drinking water [63,65].

CDI (mg/kg-Day) = [Cdw (mg/L) × IR (L/Day) × EF (Day/Year) × ED (Years)]/[BW (kg) × AT (Days)](1)

HQ = [CDI (mg/kg-Day)]/[RfD (mg/kg-Day)](2)

LCR = [CDI (mg/kg-Day)] × [SF (mg/kg-Day)^−1^] (3)

Here:

CDI = chronic daily intake (mg/kg-day);

Cdw = metal concentration in water (mg/L);

IR = water ingestion rate (1.996 L/day; questionnaire survey);

EF = exposure frequency (365 day/year [3,63]);

ED = exposure duration (74 years [3]);

BW = body weight (63.193 kg; questionnaire survey);

AT = average time (27,010 days [3,63]).

The upper bound range of lifetime cancer risk (LCR) to an individual is 10^−4^ to 10^−6^. Most highly exposed populations should not exceed 10^−5^ risk level; however, if cancer risk value is greater than 10^−5^, then action must be taken to protect the populations [65]. Similarly, any value of hazard quotient (HQ) ≥ 1 should be taken seriously to avoid non-carcinogenic risks to humans [3,65]. Therefore, the chronic daily intake (CDI) of Cd and Cr at Langat Basin, Malaysia was calculated through Equation (1). The HQ and LCR values were calculated according to Equations (2) and (3), respectively, using the RfD (oral reference dose) of Cd (5 × 10^−4^ mg/kg-day) [66] and Cr (3 × 10^−3^ mg/kg-day) [67], as well as the slope factor value of Cr (5 × 10^−1^ (mg/kg-day) [67].

### 2.3. Household Questionnaire Survey

According to the latest population census by the Department of Statistic Malaysia, the total number of households in the Langat River Basin is 1,494,865 [68]. A 402-household questionnaire survey was conducted at the basin using Equation (IV) [69,70] to obtain the average daily drinking water intake by the population in the basin. Additionally, the body weight of household members was used to calculate the CDI of Cd and Cr ingestion through drinking water.

*n* = [*N*/(1 + *N* (e)^2^)] (4)

Here: 

*n* = sample size;

*N* = population size;

e = level of precision (0.05 at 95% confidence level).

### 2.4. Prediction Model of Metal Concentration in Water 

Time series (2005–2014) monthly Langat River water quality data for Cd and Cr were provided by the Department of Environment (DOE) Malaysia. Therefore, the time series auto regression moving average statistical analysis was applied to estimate Cd and Cr concentration models in January 2020 on the basis of DOE (2005–2014) and laboratory data (2015–2016) [71,72,73]. Moreover, the assumptions of time series data analysis were fulfilled with a significant augmented Dickey–Fuller (ADF) unit root test for these metals at 0.01 level. Assumptions were also confirmed through autocorrelation (PACF) and partial autocorrelation (PACF) plots at 95% confidence level.

## 3. Results and Discussions 

### 3.1. Metal Concentrations in Drinking Water Supply Chain 

Concentrations of Cd and Cr in the drinking water supply chain (Table 1) at the Langat River basin, Malaysia, were within the drinking water quality standards of Ministry of Health Malaysia (MOH), World Health Organization (WHO), USEPA, and European Commission (EC). The skewness (<2) and kurtosis (<2) analyses of Cd and Cr concentrations in the river, treated, and tap water indicated normal distribution of the data, except in the household (HH) filtered water data of Cr because the kurtosis value was >4. 

The maximum high concentration of Cd (34.3 × 10^−4^ mg/L) in the Langat River might be due to the natural weathering of Cd from the zinc ores such as sphalerite (ZnS) or Cd minerals such as greenockite [79] in the Titiwangsa Granite Hill Range of the basin. The point sources of pollution from sewage treatment plant effluent also attributed high concentration of Cd. Similarly, waste dumping in the river, runoff from landfills, and industrial waste from the metal finishing process at Bukit Tempoi might have contributed to high Cd concentration in the Langat River. Accordingly, the maximum concentration of Cr (12.2 × 10^−4^ mg/L) in the Langat River indicated pollution in the mid-stream of the river basin from metal finishing industries such as electroplating, etching, and preparation of metal components for various industries [6,80]. Similarly, corrosion inhibitors, pigments from industrial effluents, and lithogenic sources contributed to high concentrations of Cr in Langat River [10,81].

**Figure 2:**
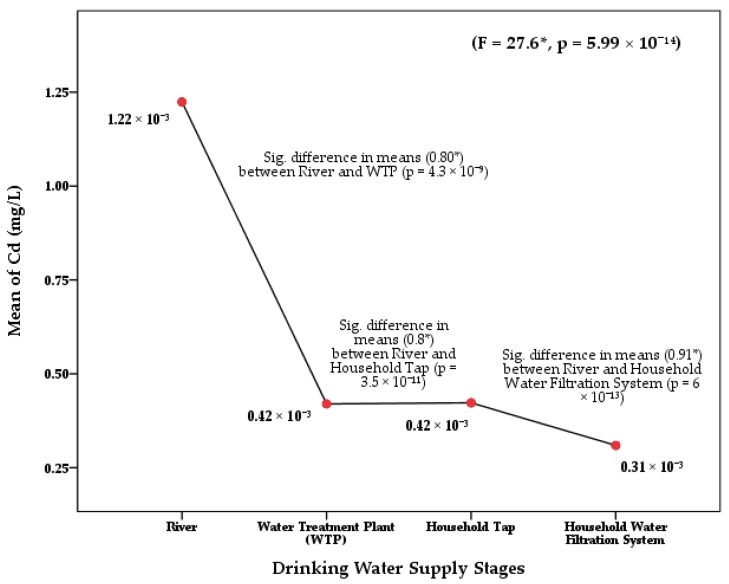
Difference in means of Cd concentrations in the drinking water supply chain at the Langat River Basin, Malaysia. Note: * significant at a 95% confidence level (Table A2).

**Figure 3:**
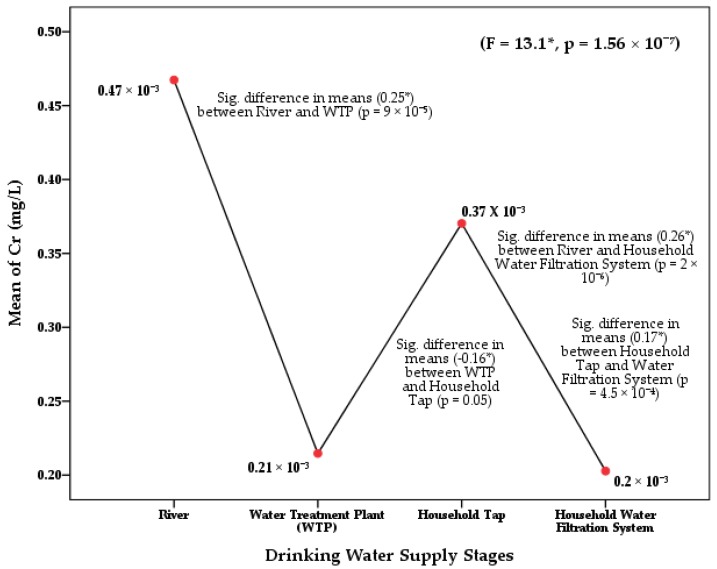
Difference in means of Cr concentrations in the drinking water supply chain at the Langat River Basin, Malaysia. Note: * significant at a 95% confidence level (Table A2).

**Figure 4:**
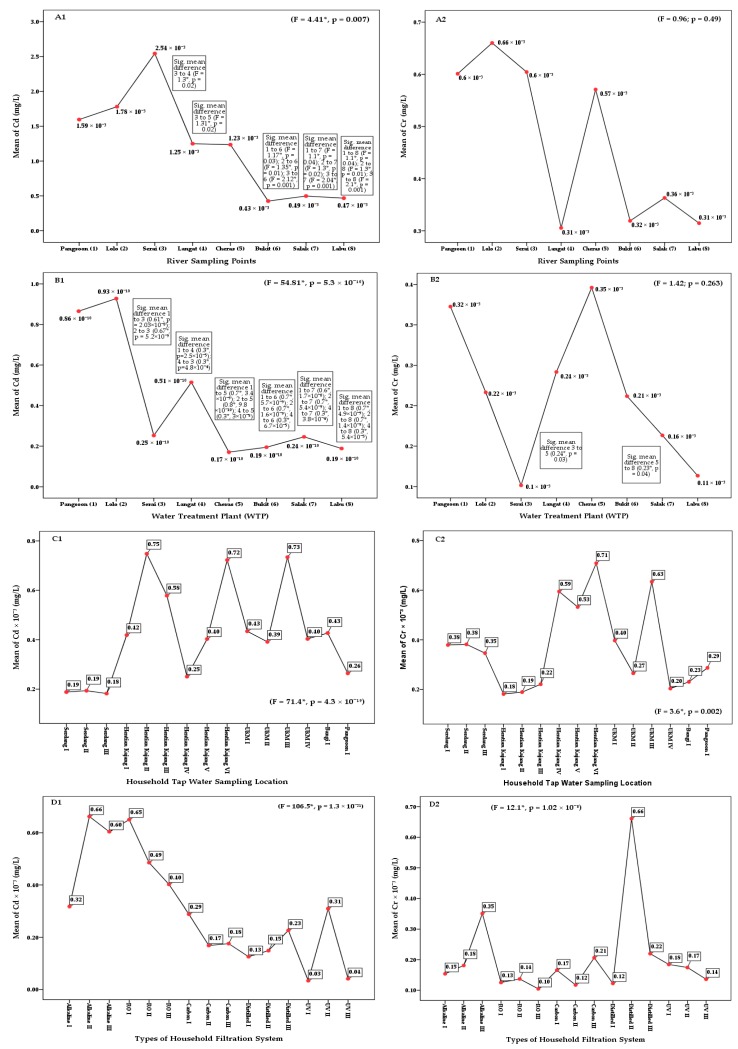
Cd and Cr concentration differences among the sampling points ((**A1**,**A2**) river, (**B1**,**B2**) water treatment plant (WTP), (**C1**,**C2**) tap water, (**D1**,**D2**) household (HH) filtered water). Note: * one-way ANOVA and least significant difference (LSD) post hoc test is significant at a 95% confidence level.

The one-way ANOVA of Cd (*F* = 27.6; *p* = 5.99 × 10^−14^) and Cr (*F* = 13.1; *p* = 1.56 × 10^−7^) in the Langat River Basin found significant differences at 0.05 confidence level among the four stages of drinking water supply chain (Table A1). The least significant difference (LSD) of the post hoc test also found significant mean differences of Cd concentration between river water and water treatment plants (*p* = 4.3 × 10^−9^), tap water (*p* = 3.5 × 10^−11^), and HH filtered water (*p* = 6 × 10^−13^) at 95% confidence interval (Figure 2). Similarly, significant differences were found in the concentration of Cr between river water and treatment plants (*p* = 9 × 10^−5^) and HH filtered water (*p* = 2 × 10^−6^) (Figure 3). Moreover, significant differences of Cd and Cr concentrations were also observed among the river water sampling points, as well as among the WTPs, tap water, and HH filtered water at a 95% confidence level (Figure 4).

The mean dissolved concentration of Cd in the supply water of the basin was estimated as being 0.42 × 10^−3^ ± 0.19 × 10^−3^ mg/L (Table 2) and was within the drinking water quality standard proposed by MOH and WHO (0.003 mg/L). The highest concentrations of dissolved Cd was observed at the location Hentian Kajang II (0.75 × 10^−3^ ± 0.02 × 10^−3^ mg/L), followed by the location Universiti Kebangsaan Malaysia (UKM) III 0.73 × 10^−3^ ± 0.04 × 10^−3^ mg/L. The high concentration of dissolved Cd in the water distribution system might have been due to corrosion in galvanized (i.e., zinc-coated) pipelines or cadmium-containing solders in fittings and taps. Hence, the leaching of Cd from galvanized pipes occurred because of the presence of Cd and lead (Pb) impurities in the zinc [82] of galvanized pipe along with the residence time of low pH water from the use of lime in water treatment [17]. 

Similarly, the mean concentration of dissolved Cr in the supply water of the basin (0.37 × 10^−3^ ± 0.21 × 10^−3^ mg/L) was lower than the maximum limit of drinking water quality standard proposed by the MOH, WHO, and EC (0.5 mg/L). The highest concentrations of dissolved Cr were recorded at Hentian Kajang VI (0.71 × 10^−3^ ± 0.41 × 10^−3^ mg/L) and Universiti Kebangsaan Malaysia (UKM) III (0.63 × 10^−3^ ± 0.02 × 10^−3^ mg/L). The high concentration of dissolved Cr at Hentian Kajang and UKM might have been due to corrosion of Cr in the steel pipes (steel alloy and chromium) of the drinking water distribution system [83,84,85,86]. Moreover, the stagnant water period in the water distribution system was also an important factor to increase the concentration of dissolved Cr in supply water [87]. 

Accordingly, the high concentration of Cd at Alkaline II (0.66 × 10^−3^ ± 0.08 × 10^−3^ mg/L) and Reverse Osmosis (RO) I filtered water (0.65 × 10^−3^ ± 0.01 × 10^−3^ mg/L) might have been due to microorganism growth on the cartridge. Irregular cleaning activities can lead to inorganic ion deposition on the cartridge [88,89,90,91,92]. Leaching of ions from the cartridge contributed to high concentrations of trace metals (e.g., Cd) in drinking water. However, the mean concentration of dissolved Cd (0.31 × 10^−3^ ± 0.21 × 10^−3^ mg/L) in HH filtration water at the basin was lower than the drinking water quality standards of 0.003 mg/L proposed by the MOH and WHO and 0.5 mg/L proposed by the USEPA and EC. However, the RO vendor machine at Johor, Malaysia, found a lower concentration of Cd (0.08 × 10^−3^ ± 0.03 × 10^−3^ mg/L) and Cr (0.39 × 10^−3^ ± 0.09 × 10^−3^ mg/L) in the filtered water [93].

The high dissolved concentrations of Cr in the Distilled II (0.66 × 10^−3^ ± 0.003 × 10^−3^ mg/L) and Alkaline III (0.35 × 10^−3^ ± 0.13 × 10^−3^ mg/L) filtered waters might have been due to corrosion of galvanized iron pipes linked to steel pipes at the end of the reticulation system along with stagnant water time within the filter. Moreover, rust inside distilled filters and a lack of cleaning activities also contributed to high concentrations of Cr in the drinking water. However, the mean concentration of dissolved Cr (0.2 × 10^−3^ ± 0.15 × 10^−3^ mg/L) in the HH filtered water at the basin was below the maximum limit of the drinking water quality standard of Cr (0.50 mg/L) proposed by the MOH, WHO, and EC.

### 3.2. Prediction Model of Metal Concentrations in Drinking Water Supply Chain 

**Figure A1:**
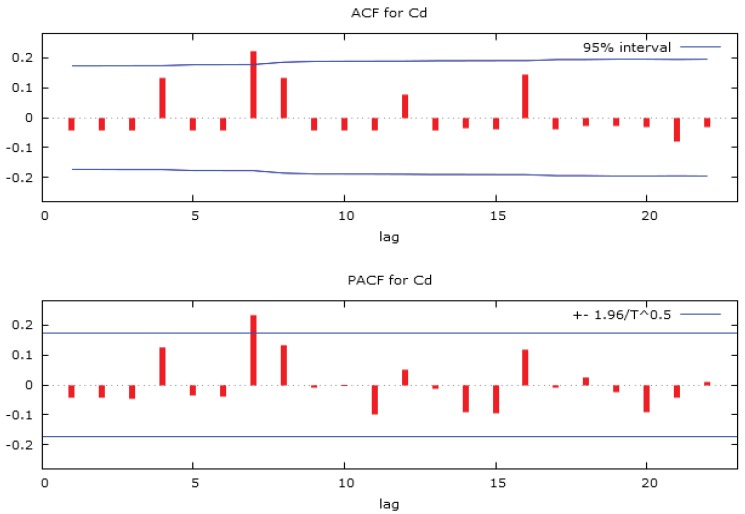
Auto correlation (ACF) and partial auto correlation (PACF) correlogram for Cd concentration.

**Figure A2:**
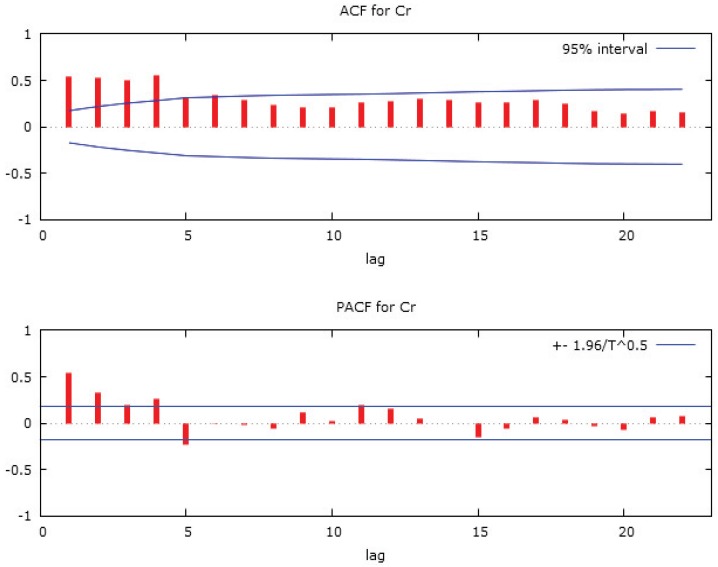
Auto correlation (ACF) and partial auto correlation (PACF) correlogram for Cr concentration.

The time series data of Cd and Cr concentrations in Langat River complied with the time series data analysis at 99% confidence interval. The compliance of time series data analysis was based on the significant augmented Dickey–Fuller (ADF) unit root test for Cd and Cr at 0.05 level (Table A3). The ADF unit root test of Cr with constant was not significant (*p* = 0.65) at the 0.05 level; however, the ADF unit root test of Cr with constant (i.e., considering Cr trend) was significant (*p* = 7.17 × 10^−2^) at the 0.05 level. Similarly, the autocorrelation (ACF) plots based on the differences in Cd and Cr concentrations showed significant autocorrelation only at Lag 1, although the ADF unit root test of Cd and Cr data remained static at a 95% confidence level. Similarly, the partial autocorrelation (PACF) plots based on the differences Cd and Cr concentrations with a 95% confidence band showed that the autocorrelation was only significant at Lag 1 and Lag 2 (Figure A1 and Figure A2). Therefore, this study used a monthly (2005–2020) auto regression model to estimate the Lag effects on Cd (Table A4) and Cr (Table A5) concentrations in the drinking water supply chain of the Langat River Basin. The impact of the prior three months (i.e., identified Lags in Table A6) had significant influence on the Cd concentration trend in Langat River after 2016. The predicted Cd concentration considering the influences of environmental parameters (i.e., water flow, rainfall, and temperature) was also similar to the determined Cd concentration in 2015. Similarly, the impact of prior month (i.e. identified Lag in Table A7) had significant influence on the Cr concentration trend in Langat River after 2016.

**Figure 5:**
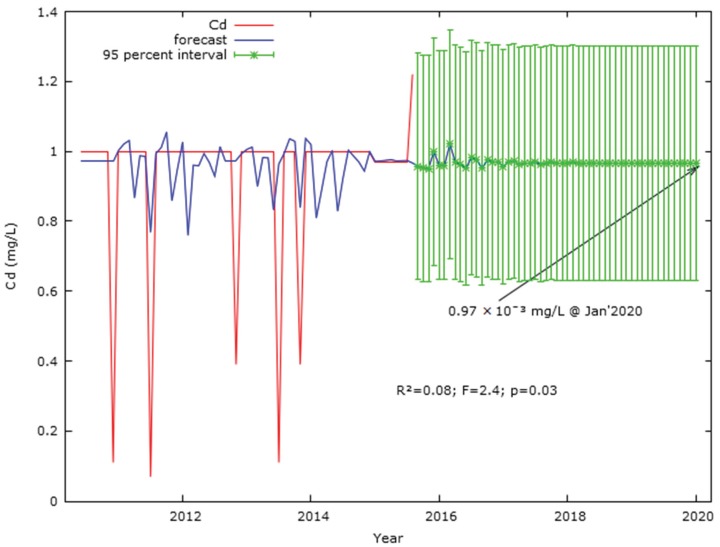
Forecast of Cd (mg/L) concentration in Langat River on the basis of auto-generative moving average from January 2005 to January 2020. Note: *Y*-axis refers to Cd concentration in mg/L and explains the predicted Cd concentration along with the range of percentile to validate the predicted trend.

**Figure 6:**
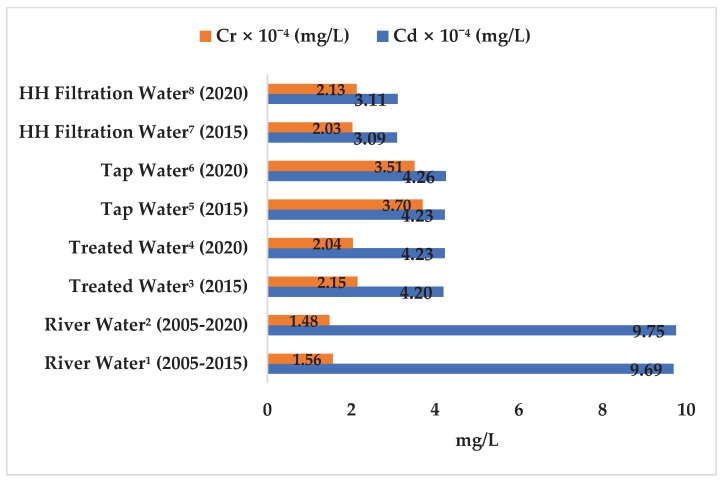
Forecast of Cr (mg/L) concentration in Langat River on the basis of auto-generative moving average from January 2005 to January 2020. Note: *X*-axis refers to Cd and Cr concentrations in mg/L. Percentage change of Cd (0.63%) and Cr (−5.04%) concentration was based on the difference of concentration between 2005–2015 and 2020. ^1^ Determined concentrations were based on the time series data (2005–2014) and laboratory results (2015) of this study. ^2^ Predicted concentrations were obtained from the auto-generative moving average forecast model. ^3,5,7^ Laboratory results of this study. ^4,6,8^ Predicted concentrations in treated, tap, and household filter water were calculated on the basis of the percentage change of metal concentrations in the river. HH refers to household.

**Figure A3:**
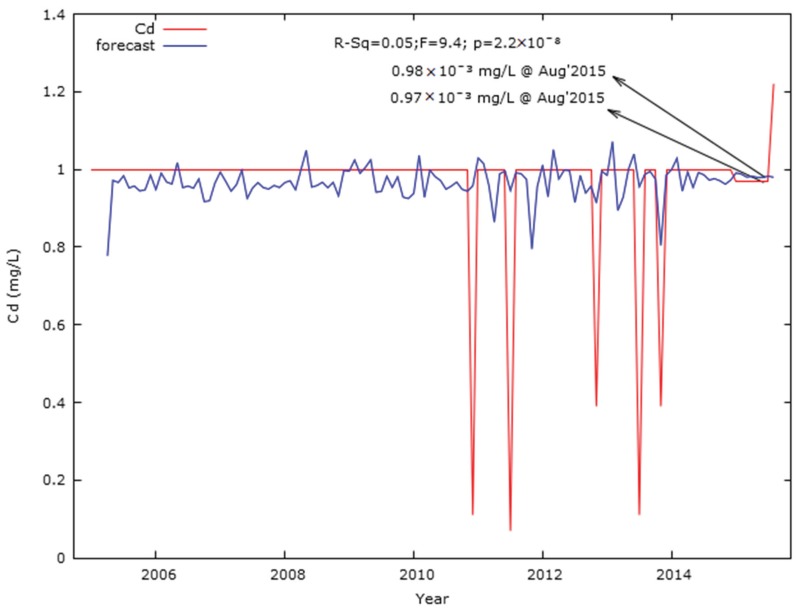
Forecast of Cd (mg/L) concentration considering control variables in Langat River on the basis of auto-regressive analysis from 2005 to 2015. Note: *Y*-axis refers to Cd concentration in mg/L and explains the determined and predicted Cd concentration considering the influences of environmental parameters.

Lag 1 to Lag 7 effects in the auto-regressive Cd model (Table A4) suggested that the consecutive prior seven months had a significant impact on the Cd concentration of the current month in Langat River, where Lag 7 (*t* = 2.32; *p* = 0.02) was significant at the 0.05 level. In addition, the sixth-order auto-generative coefficient weight 0.21 (*t* = 2.23; *p* = 0.03) was significant at the 0.05 level to have an autocorrelation-free Cd concentration forecast model, because Lag 7 in both ACF and PACF crossed the 95% interval line, indicating the existence of autocorrelation. Therefore, the auto-regressive moving average of Cd concentration based on the data from January 2005 to August 2015 forecasted 9.7 × 10^−4^ mg/L in January 2020 (Figure 5) and a mean Cd concentration of 9.75 × 10^−4^ ± 1.33 × 10^−4^ mg/L during 2005–2020 (Figure 6). Moreover, the predicted Cd concentration (9.7 × 10^−4^ mg/L) in 2020 was a little bit higher than the mean Cd concentration (9.69 × 10^−4^ ± 1.57 × 10^−4^) mg/L in Langat River during 2005–2015 (Figure 6). The predicted Cd concentration (9.7 × 10^−4^ mg/L) at January 2020 in Langat river was significant (*R^2^* = 0.08; *F* = 2.4; *p* = 0.03) at a 95% confidence interval. Similarly, the forecast of Cd concentration (9.8 × 10^−4^ mg/L) in August 2015 was similar to the real concentration of Cr 9.7 × 10^−4^ mg/L in August 2015 in Langat River considering the influence of significant environmental parameters (Figure A3). Moreover, the concentration of Cd in the Langat River was influenced by the Cd concentration of the prior 3 months (*t* = −2.37; *p* = 0.02; Table A6) and the model was significant (*R^2^* = 0.05; *F* = 9.4; *p* = 2.2 × 10^−8^; Figure A3) at the 0.05 level. 

**Figure 7:**
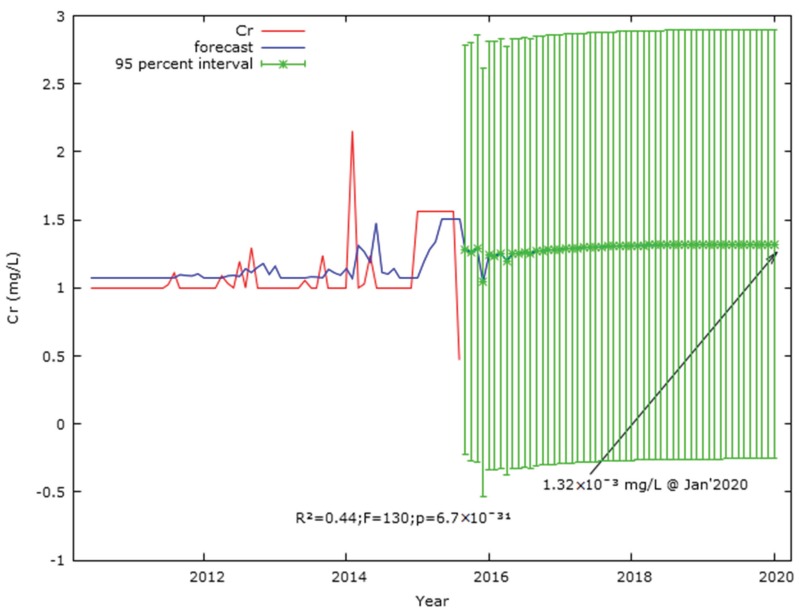
Forecast of Cr (mg/L) concentration in Langat River on the basis of auto generative moving average from January 2005 to January 2020. Note: *Y*-axis refers to Cr concentration in mg/L and explains the predicted Cr concentration along with the range of percentile to validate the predicted trend.

**Figure A4:**
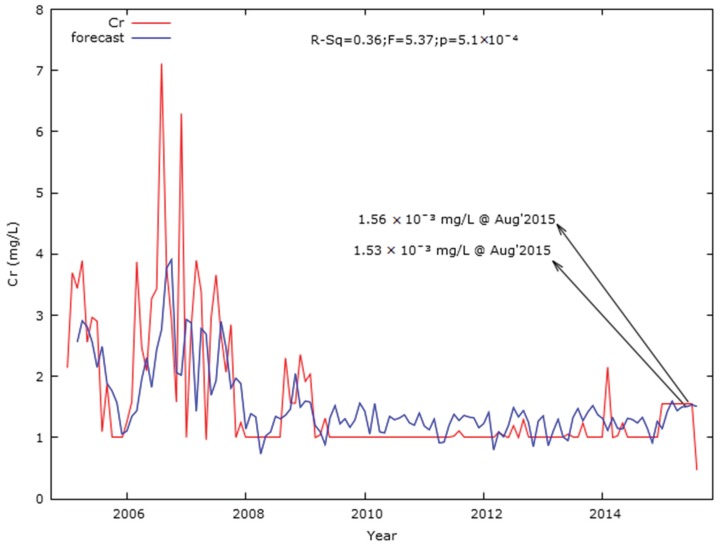
Forecast of Cr (mg/L) concentration considering control variables in Langat River on the basis of auto-regressive analysis from 2005 to 2015. Note: *Y*-axis refers to Cr concentration in mg/L and explains the determined and predicted Cr concentration considering the influences of environmental parameters.

The significant Lag 1 (*t* = 3.1; *p* = 0.002) and Lag 2 (*t* = 9.63; *p* = 1.25 × 10^−16^) effects at the 0.05 level in the auto-regressive Cr model (Table A5) suggested that the impact of the prior 2 months had significant effects on the Cr concentration in Langat River. Similarly, the second order auto-generative coefficient weight of −0.47 (*t* = −5.998; *p* = 2.13 × 10^−8^; Table A5) was significant at the 0.05 level to have an autocorrelation-free Cr concentration forecast model; the ACF and PACF correlogram of the initial few Lags crossed the 95% interval line, indicating the existence of autocorrelations. Therefore, the auto-generative moving average of Cr concentration based on the data from January 2005 to August 2015 forecasted 1.32 × 10^−3^ mg/L in January 2020 (Figure 7) as well as a mean Cr concentration of 1.48 × 10^−3^ ± 8.84 × 10^−4^ mg/L during 2005–2020. Moreover, the predicted Cr concentration (1.32 × 10^−3^ mg/L) in January 2020 was lower than the mean Cr concentration (1.56 × 10^−3^ ± 1.05 × 10^−3^ mg/L) in Langat River during 2005–2015. However, the predicted Cr concentration (1.32 × 10^−3^ mg/L) at January 2020 in Langat river was significant (*R^2^* = 0.44; *F* = 130.28; *p* = 6.7 × 10^−31^; Figure 7) at a 95% confidence level. Similarly, considering the control variables such as water flow, rainfall, and temperature, the Cr concentration in Langat River at 2015 was significantly influenced by the concentration of prior two months (Lag 2, *t* = 3.6744; *p* = 0.0004; Table A7). The model was significant (*R^2^* = 0.36; *F* = 5.37; *p* = 5.1 × 10^−4^; Figure A4) at the 0.05 level and the forecasted and real concentrations of Cr in the Langat River were almost similar.

### 3.3. Prediction Model of Metal Concentrations in Drinking Water Supply Chain 

**Figure 8:**
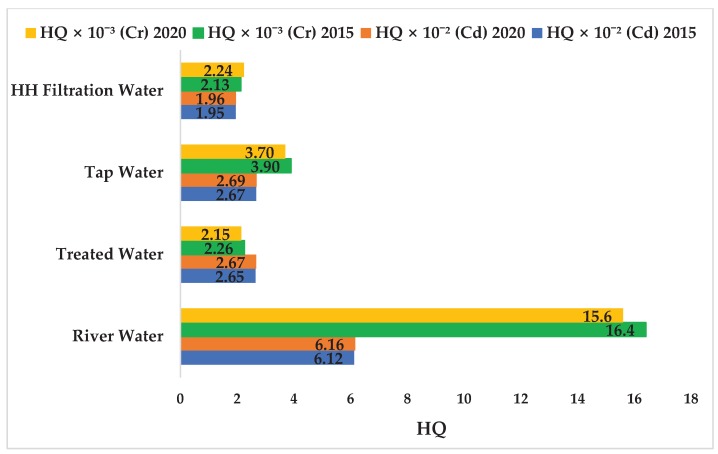
Non-carcinogenic risk values of Cd and Cr ingestion via drinking water in Langat River Basin, Malaysia. Note: *X*-axis refers to the non-carcinogenic value of hazard quotients (HQ), and HQ value < 1 is safe. River: HQ (Cd) 2015, *t* = 69.88, *p* = 3.2 × 10^−103^ *; HQ (Cd) 2020, *t* = 98.97, *p* = 7.1 × 10^−159^ *; HQ (Cr) 2015, *t* = 16.83, *p* = 3.81 × 10^−34^ *; HQ (Cr) 2020, *t* = 22.41, *p* = 5.84 × 10^−54^ *; treated water: HQ (Cd) 2015, *t* = 6.714, *p* = 7.55 × 10^−7^ *; HQ (Cr) 2015, *t* = 7.670, *p* = 8.77 × 10^−8^ *; tap: HQ (Cd) 2015, *t* =14.58, *p* = 1.88 × 10^−18^ *; HQ (Cr) 2015, *t* = 11.64, *p* = 5 × 10^−15^ *; HH (household) filtration: HQ (Cd) 2015, *t* = 9.99, *p* = 6.72 × 10^−13^ *; HQ (Cr) 2015, *t* = 9.35, *p* = 4.12 × 10^−12^ *; * significant at 95% level.

Cd concentration in the drinking water supply chain at Langat Basin both in 2015 and 2020 were within the maximum limit of the drinking water quality standard of the MOH (0.003 mg/L), WHO (0.003 mg/L), and USEPA (0.0022 mg/L). Cadmium concentration (3.11 × 10^−4^ mg/L) in HH filtration water in 2020 (Figure 6) was also well below the maximum tolerable daily intake of Cd through drinking water (8.3 × 10^−4^ mg/L) [37]. Therefore, Cd ingestion through HH filtration water in the Langat Basin posed no health risk because the HQ (2.67 × 10^−2^ ± 1.23 × 10^−2^ mg/L and 2.69 × 10^−2^ mg/L in 2015 and 2020, respectively; Figure 8) were significantly within the safe limit (i.e., HQ < 1 at 95% confidence level). 

**Figure 9:**
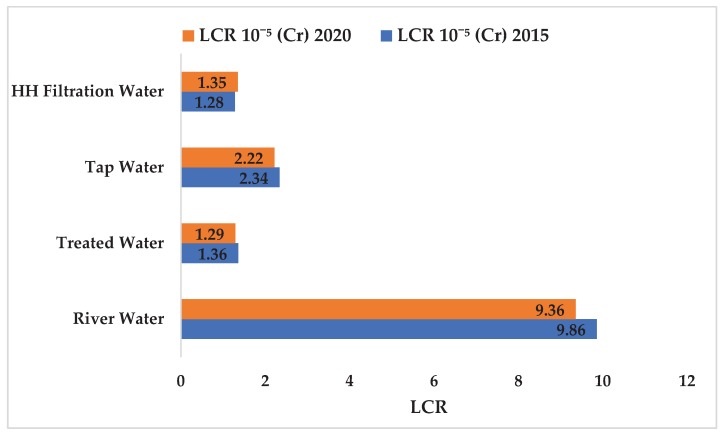
Carcinogenic risk values of Cr ingestion via drinking water in Malaysia. Note: *X*-axis refers to the carcinogenic lifetime cancer risk (LCR) value and LCR value < 10^−5^ is acceptable. River: LCR (Cr) 2015, *t* = 16.83, *p* = 3.81 × 10^−34^ *; LCR (Cr) 2020, *t* = 22.41, *p* = 5.84 × 10^−54^ *; treated water: LCR (Cr) 2015, *t* = 7.670, *p* = 8.77 × 10^−8^ *; tap: LCR (Cr) 2015, *t* = 11.64, *p* = 5 × 10^−15^ *; HH (household) filtration: LCR (Cr) 2015, *t* = 9.35, *p* = 4.12 × 10^−12^ *; * significant at 95% level. LCR = lifetime cancer risk.

Accordingly, Cr concentration in the drinking water supply chain in Langat Basin in 2015 and 2020 were within the safe limit of the drinking water quality standard of the MOH (0.05 mg/L), WHO (0.05 mg/L), and USEPA (0.011 mg/L). The concentration of Cr (2.13 × 10^−4^ mg/L) in HH filtration water in 2020 were predicted in this study (Figure 6), however, the maximum tolerable daily intake of Cr through drinking water for humans has yet to be fixed [94]. Hence, Cr ingestion through HH filtration water in Langat Basin showed no potential non-carcinogenic human health risk (2.13 × 10^−3^ ± 1.55 × 10^−3^ mg/L and 2.24 × 10^−3^ mg/L in 2015 and 2020, respectively; Figure 8) because the values were within the safe limit (i.e., HQ < 1). Accordingly, the LCR values of Cr ingestion through HH filtration water (1.28 × 10^−5^ ± 9.29 × 10^−6^ mg/L and 1.35 × 10^−5^ mg/L in 2015 and 2020, respectively; Figure 9) were within the safe limit because the LCR values were not greater than ≥1 × 10^−5^ mg/L at a 95% confidence level. 

All eight water treatment plants (WTPs) in Langat Basin follow the conventional water treatment method. However, this conventional method was unable to fully remove trace metals from the treated water mainly because of frequent changes in turbidity in Langat River [3]. For instance, Frey [95] reported that a conventional coagulation method can remove Cr (III) from water; however, it cannot remove Cr (VI). Similarly, Brandhuber [96] reported that the total Cr removal varied between 40% and 100% with the conventional method because Cr (VI) cannot be removed by alum or ferric coagulation or by lime softening. Therefore, a two-layer water filtration system should be introduced in the Langat Basin because treated water contamination in the long pipeline was evident between WTPs and households; additionally, the conventional method was unable to fully remove metals from treated water. Hence, the reverse osmosis membrane technology can be appropriate to install in a kitchen tap of the household managed by the water billing agency because it can remove more than 90% of trace metals [97].

## 4. Recommendations 

A two-layer water filtration system at the basin should be introduced to achieve the SDG target 6.1 of obtaining safe drinking water supply before 2030. Because the traditional coagulation method is unable to completely remove metals from treated water, and treated water contamination in the long pipeline was evident in between WTPs and households, a reverse osmosis filtration system with the capacity to remove more than 90% of metals could be installed at the kitchen tap of household. The installed reverse osmosis filtration system at household could be managed by the water billing agency and a less-expensive pond sand filtration at the treatment plants could be maintained. Furthermore, in managing the drinking water, the proactive leadership roles of local authority would be appropriate to enable the PENTA-HELIX (i.e. consists of five types of stakeholders such as public, private, academia, non-governmental organization and community) partnership model to bring public, business, academia, NGO (non-governmental organization), and community sectors into the same multi-stakeholder platform.

## 5. Conclusions

Cadmium and chromium concentrations in the drinking water supply chain at Langat Basin were within the drinking water quality standard of the Ministry of Health Malaysia and WHO. Moreover, Cd and Cr ingestion through household filtration water in the Langat Basin poses no health risk because the hazard quotients (HQ) of Cd and Cr were significantly within the safe limit in 2015 and 2020. Similarly, the LCR (lifetime cancer risk) value of Cr ingestion through household filtration water was within the safe limit in 2015 and 2020. However, high concentrations of these metals have been found in the household tap water mainly because of contamination in the water distribution pipeline. The age-old water distribution pipelines in between water treatment plants and households as well as the old water reticulation systems at the households in the Langat Basin might have attributed to Cd and Cr concentrations in the household tap water. Similarly, the irregular cleaning activities of the household water filtration systems might have also attributed to the Cd and Cr concentrations in the drinking water. 

## Figures and Tables

**Table 1:** Mean Cd and Cr concentrations (mg/L) in drinking water at Langat River Basin, Malaysia (2015).

Sample	Metal	Range	Mean	Skewness	Kurtosis	MOH ^1^	USEPA ^2^	EC ^3^
River water	Cd (mg/L)	3.9 × 10^−4^–34.3 × 10^−4^	12.2 × 10^−4^ ± 3.8 × 10^−4^	1.03	0.13	0.003	0.00072	0.0022
	Cr (mg/L)	1.2 × 10^−4^–12.2 × 10^−4^	4.7 × 10^−4^ ± 2.7 × 10^−4^	1.33	1.90	0.05	0.011	–
Treated water		1.2 × 10^−4^–9.9 × 10^−4^	4.2 × 10^−4^ ± 3.1 × 10^−4^	0.91	−0.84	0.003	0.005 ^4^	0.003 ^5^
		0.2 × 10^−4^–5.3 × 10^−4^	2.1 × 10^−4^ ± 1.4 × 10^−4^	0.95	0.14	0.05	0.1 ^4^	0.05 ^5^
Tap water		1.3 × 10^−4^–7.7 × 10^−4^	4.2 × 10^−4^ ± 1.9 × 10^−4^	0.49	−0.80	0.003	0.005 ^4^	0.003 ^5^
		1.0 × 10^−4^–9.5 × 10^−4^	3.7 × 10^−4^ ± 2.1 × 10^−4^	1.08	0.76	0.05	0.14	0.05 ^5^
HH ^6^ filtration		0.3 × 10^−4^–7.4 × 10^−4^	3.1 × 10^−3^ ± 2.1 × 10^−3^	0.49	−0.90	0.003	0.005 ^4^	0.003 ^5^
		0.5 × 10^−4^–6.6 × 10^−4^	2 × 10^−3^ ± 1.5 × 10^−3^	2.00	4.31	0.05	0.1 ^4^	0.05 ^5^

Note: ^1^ Raw Water Quality Standard proposed by Ministry of Health Malaysia [74]; ^2^ Criteria Continuous Concentration by United States Environmental Protection Agency [75]; ^3^ Annual Average proposed by European Commission [76]; ^4^ Regulated Drinking Water proposed by United States Environmental Protection Agency [77]; ^5^ Guidelines for Drinking Water Quality proposed by World Health Organization [78]. ^6^ HH filtration refers to household’s filtered water sample.

**Table 2:** Cd × 10^−3^ and Cr × 10^−3^ concentrations (mg/L) in drinking water supply chain at Langat River Basin, Malaysia (2015).

Water Sampling Locations	River		Water Treatment Plant		Filter (Household Locations)	HH ^1^ Tap		HH ^2^ Filtration	
	Cd (mg/L)	Cr (mg/L)	Cd (mg/L)	Cr (mg/L)		Cd (mg/L)	Cr (mg/L)	Cd (mg/L)	Cr (mg/L)
Pangsoon	1.60 ± 0.66	0.60 ± 0.56	0.87 ± 0.12	0.32 ± 0.21	Alkaline I (Serdang I)	0.19 ± 0.06	0.38 ± 0.12	0.32 ± 0.002	0.15 ± 0.09
Lolo	1.78 ± 1.43	0.66 ± 0.36	0.93 ± 0.06	0.22 ± 0.2	Alkaline II (Serdang II)	0.19 ± 0.03	0.38 ± 0.12	0.66 ± 0.08	0.18 ± 0.09
Serai	2.54 ± 0.02	0.60 ± 0.04	0.25 ± 0.09	0.1 ± 0.02	Alkaline III (Serdang III)	0.18 ± 0.02	0.35 ± 0.12	0.6 ± 0.02	0.35 ± 0.13
Langat	1.25 ± 0.09	0.31 ± 0.14	0.51 ± 0.1	0.24 ± 0.16	RO ^3^ I (Hentian Kajang I)	0.42 ± 0.01	0.18 ± 0.03	0.65 ± 0.01	0.13 ± 0.04
Cheras	1.23 ± 0.73	0.57 ± 0.32	0.17 ± 0.05	0.35 ± 0.13	RO ^3^ II (Hentian Kajang II)	0.75 ± 0.02	0.19 ± 0.01	0.49 ± 0.03	0.14 ± 0.09
Bukit	0.43 ± 0.03	0.32 ± 0.12	0.19 ± 0.02	0.21 ± 0.07	RO ^3^ III (Hentian Kajang III)	0.58 ± 0.08	0.22 ± 0.08	0.4 ± 0.01	0.1 ± 0.02
Salak	0.50 ± 0.04	0.36 ± 0.02	0.25 ± 0.05	0.16 ± 0.01	Carbon I (Hentian Kajang IV)	0.25 ± 0.04	0.59 ± 0.2	0.29 ± 0.04	0.17 ± 0.09
Labu	0.47 ± 0.04	0.31 ± 0.12	0.19 ± 0.05	0.11 ± 0.03	Carbon II (Hentian Kajang V)	0.40 ± 0.03	0.53 ± 0.08	0.17 ± 0.08	0.12 ± 0.03
Mean	1.22 ± 0.38	0.47 ± 0.21	0.42 ± 0.31	0.21 ± 0.14	Carbon III (UKM II)	0.39 ± 0.03	0.27 ± 0.25	0.18 ± 0.01	0.21 ± 0.01
					Distilled I (UKM III)	0.73 ± 0.04	0.63 ± 0.02	0.13 ± 0.01	0.12 ± 0.01
					Distilled II (Hentian Kajang VI)	0.72 ± 0.07	0.71 ± 0.41	0.15 ± 0.02	0.66 ± 0.003
					Distilled III (UKM I)	0.43 ± 0.03	0.4 ± 0.14	0.23 ± 0.04	0.22 ± 0.07
					UV I (Bangi I)	0.43 ± 0.01	0.23 ± 0.150	0.03 ± 0.01	0.18 ± 0.01
					UV II (UKM IV)	0.4 ± 0.03	0.2 ± 0.04	0.31 ± 0.02	0.17 ± 0.12
					UV III (Hentian Kajang VII)	0.26 ± 0.03	0.29 ± 0.004	0.04 ± 0.01	0.14 ± 0.04
					Mean	0.42 ± 0.19	0.37 ± 0.21	0.31 ± 0.21	0.2 ± 0.15

Note: ^1^ HH refers to household. ^2^ HH filtration refers to filtered water at household. ^3^ RO refers to reverse osmosis (household water filtration system).

## Appendix A

**Table A1:** One-way ANOVA of Cd and Cr concentrations in drinking water supply chain, Langat Basin.

Water Quality Parameter		Sum of Squares			Mean Square		
	*N*	Between Groups	Within Groups	Total	Between Groups	Within Groups	*F*
Cd	138	14.6	23.6	38.1	489	0.2	27.6 * (*p* = 5.99 × 10^−14^)
Cr	138	1.5	5.1	6.6	0.5	0.04	13.1 * (*p* = 1.56 × 10^−7^)

* Significant at 0.05 level.

**Table A2:** Multiple comparisons of Cd (mg/L) and Cr (mg/L) means based on Tukey HSD (Honestly Significant Difference) test in drinking water supply chain at Langat River Basin, Malaysia.

Dependent Variable	(I) Drinking Water Supply Stages	(J) Drinking Water Supply Stages	Mean Difference (I-J)	Standard Error	Significance (*p*)	95% Confidence Interval	
						Lower Bound	Upper Bound
Cd (mg/L)	River	Water treatment plant (WTP)	0.80 *	0.12	4.3 × 10^−9^	0.49	1.12
		Household tap	0.80 *	0.11	3.5 × 10^−11^	0.53	1.08
		Household water filtration system	0.91 *	0.11	6 × 10^−13^	0.64	1.19
	Water treatment plant (WTP)	River	−0.80 *	0.12	4.3 × 10^−9^	−1.12	−0.49
		Household tap	0.003	0.11	1	−0.28	0.27
		Household water filtration system	0.11	0.11	0.73	−0.17	0.39
	Household tap	River	−0.80 *	0.11	3.5 × 10^−11^	−1.08	−0.53
		water treatment plant (WTP)	0.003	0.11	1	−0.27	0.28
		Household water filtration system	0.11	0.09	0.58	−0.12	0.34
	Household water filtration system	River	−0.91 *	0.11	6 × 10^−13^	−1.19	−0.64
		water treatment plant (WTP)	−0.11	0.11	0.73	−0.39	0.17
		Household tap	−0.11	0.09	0.58	−0.34	0.12
Cr (mg/L)	River	Water treatment plant (WTP)	0.25 *	0.06	9 × 10^−5^	0.11	0.40
		Household tap	0.10	0.05	0.21	−0.03	0.23
		Household water filtration system	0.26 *	0.05	2 × 10^−6^	0.14	0.39
	Water treatment plant (WTP)	River	−0.25 *	0.06	9 × 10^−5^	−0.40	−0.11
		household tap	−0.16 *	0.05	0.01	−0.28	−0.03
		Household water filtration system	0.01	0.05	0.99	−0.12	0.14
	Household tap	River	−0.10	0.05	0.21	−0.23	0.03
		water treatment plant (WTP)	0.16 *	0.05	0.01	0.03	0.28
		Household water filtration system	0.17 *	0.04	4.5 × 10^−4^	0.06	0.27
	Household water filtration system	River	−0.26 *	0.05	2 × 10^−6^	−0.39	−0.14
		water treatment plant (WTP)	−0.01	0.05	0.995	−0.14	0.12
		Household tap	−0.17 *	0.04	4.5 × 10^−4^	−0.27	−0.06

Note: * The mean difference is significant at the 0.05 level.

**Table A3:** Augmented Dickey–Fuller (ADF) unit root test for auto regression.

Variable	ADF with Constant	*p*-Value	ADF with Constant and Trend	*p*-Value
Cd	−11.545	3.76 × 10^−17^ *	−11.714	2.6 × 10^−16^ *
Cr	−1.2599	6.5 × 10^1^	−3.2672	7.17 × 10^−2^ *

Note: * Significant at 0.05 level.

**Table A4:** Auto regression for Cd Model (*t* = 115).

Variables	Coefficient	Standard Error	*t*-Value	*p*-Value
Constant	1.02417	0.27284	3.754	0.0003 ***
Lag 1	−0.03538	0.09534	−0.371	0.7113
Lag 2	0.05592	0.09289	0.602	0.5485
Lag 3	−0.06715	0.09302	−0.722	0.4719
Lag 4	0.11769	0.09249	1.272	0.2060
Lag 5	−0.01826	0.09297	−0.196	0.8446
Lag 6	−0.22229	0.09283	−2.395	0.0184 ***
Lag 7	0.22085	0.09525	2.319	0.0223 ***
u (-6)	0.20617	0.09257	2.227	0.0280 **

Note: Lag (dependent variable); Lag 1 to 7 = first month to seventh month, respectively; u (-6) = auto generative weight; *** significant at 0.01 level. ** significant at 0.05 level.

**Table A5:** Auto regression for Cr model (*t* = 124).

Variables	Coefficient	Standard Error	*t*-Value	*p*-Value
Constant	0.19268	0.09610	2.005	0.0472 **
Lag 1	0.20961	0.06767	3.098	0.0024 ***
Lag 2	0.64481	0.06699	9.626	1.25 × 10−^16^ ***
u (-2)	−0.47459	0.07912	−5.998	2.13 × 10^−8^ ***

Note: Lag (dependent variable); Lag 1 = first month; Lag 2 = second month; u (-2) = auto generative weight; *** significant at 0.01 level; ****** significant at 0.05 level.

**Table A6:** Auto regression for Cd model with external variables (*t* = 124).

Variables	Coefficient	Standard Error	*t*-Ratio	*p*-Value
Constant	0.50945	0.39174	1.3005	0.1960
Water flow	−0.00073	0.00162	−0.4547	0.6502
Rainfall	−2.17297 × 10^−5^	0.00013	−0.1715	0.8641
Temperature	0.06835	0.03774	1.8109	0.0727 *
Lag 1	−0.64645	0.13525	−4.7796	5.15 × 10^−6^ ***
Lag 2	−0.40636	0.14763	−2.7525	0.0069 ***
Lag 3	−0.26401	0.11146	−2.3686	0.0195 **

Dependent variable: Cd. *** significant at 0.01 level. ** significant at 0.05 level. * significant at 0.10 level.

**Table A7:** Auto regression for Cr model with external variables (*t* = 125).

Variables	Coefficient	Std. Error	*t*-Ratio	*p*-Value
Constant	9.44345	4.79696	1.9686	0.0513 *
Water flow	−0.0129813	0.00862977	−1.5042	0.1351
Rainfall	0.000526234	0.000716295	0.7347	0.4640
Temperature	−0.321556	0.185214	−1.7361	0.0851 *
Lag 2	0.305618	0.0831747	3.6744	0.0004 ***

Dependent variable: Cr. *** significant at 0.01 level. * significant at 0.10 level.
